# Opinions and Attitudes of Anaesthesiology and Critical Care Doctors Regarding Family Presence During Resuscitation in Two Major Hospitals in Kuala Lumpur, Malaysia

**DOI:** 10.7759/cureus.94915

**Published:** 2025-10-19

**Authors:** Yu Xin Tai, Chian Yong Liu, Ismail Tan, Qurratu Aini Musthafa

**Affiliations:** 1 Anaesthesiology and Intensive Care, Hospital Canselor Tuanku Muhriz UKM (Universiti Kebangsaan Malaysia), Kuala Lumpur, MYS; 2 Anaesthesiology and Intensive Care, Hospital Kuala Lumpur, Kuala Lumpur, MYS

**Keywords:** anaesthesiology and critical care, cpr, critical care, family presence during resuscitation, family witness, fpdr, icu, intensive care

## Abstract

Family presence during resuscitation (FPDR) remains controversial despite growing acceptance in Western countries. This study explored Malaysian doctors' views on FPDR within critical care environments.

A cross-sectional survey of 169 doctors working in critical care units of Hospital Kuala Lumpur (HKL) and Hospital Canselor Tuanku Muhriz UKM (HCTM) was conducted between October 2023 and January 2024. Data analysis included descriptive statistics, chi-square tests, simple logistic regression, and multivariable logistic regression.

A total of 27.7% (n = 44) of respondents agreed to allow FPDR, with no significant association found between acceptance and demographic or professional characteristics. Major concerns included increased staff stress, potential misinterpretation of procedures, space limitations, privacy violations, emotional distress for families, difficulty stopping futile resuscitation, and litigation risks. Healthcare providers recognised FPDR's educational value and potential to promote professionalism. Opinions were divided on FPDR's impact on family participation, support, and bonding. Most did not believe FPDR reduces family anxiety. Respondents who have had personal FPDR experience were more likely to support it (OR: 8.823 (95% CI: 3.670 - 21.212), p < 0.001).

This study revealed that the majority of respondents working in critical care settings in HKL and HCTM are opposed to FPDR. Their reluctance is due to the perceived negative effects of FPDR. Experience in FPDR significantly influenced the willingness to practise FPDR.

## Introduction

Critical care is a specialised field of medicine where close monitoring and intensive medical interventions are provided to patients suffering from life-threatening illnesses or injuries. These patients are often unstable and require resuscitation should their clinical condition deteriorate. In the event of cardiac or respiratory arrest, cardiopulmonary resuscitation (CPR) is a standard of care implemented during cardiac arrest and consists of repeated chest compressions and attempts to oxygenate the lungs to restore circulation and ventilation. In the event of acute deterioration leading to resuscitation in intensive care settings, it is common to inform family members or next of kin of the situation, but they are often discouraged from witnessing the resuscitation process and hence not invited to the patient's bedside.

When family members (e.g., siblings, parents, spouses, and children) of the patient are present in the resuscitation room with the patient, it is described as “family presence during resuscitation (FPDR)” [1]. Family presence during such processes was first introduced in the literature in 1987 [2]. The arrival of the patient’s family members at the site where the resuscitation is undertaken allows them to see or have physical contact with the patient [3]. FPDR has been gaining ground in many developed countries since its first introduction in the 1980s, but remains controversial to this day [2-4].

The practice of FPDR delivers many benefits by helping with grieving and facilitating closure, providing families with understanding and recognition of the efforts made, assuring them that everything possible has been done, and also improving family-patient connectedness [5,6]. It has been suggested that FPDR offers psychological benefits to families being present during the resuscitation of loved ones [5], and it has the potential to enhance care for patients and offer benefits to patients, family members, and healthcare providers (HCPs) involved [7]. A large segment of the population desires the presence of significant others during CPR and, conversely, wants to be with loved ones during CPR [8]. Many family members view FPDR as a fundamental right, and those who have been involved reported benefits to the patient and the healthcare team [9]. Hence, with the development of more patient- and family-centred healthcare, the concept of FPDR is being emphasised, and increasing acceptance can be anticipated [10,11].

The practice of FPDR was primarily limited to Western societies, but it has begun to gain more attention in non-Western communities in recent years [12]. Several professional bodies have published recommendations or guidelines and expressed opinions in favour of FPDR, suggesting no adverse effects on resuscitation, and some possible benefits of FPDR are gradually becoming more acceptable among clinicians [1,13-15].

Systematic research has been done in many countries to explore the perception of healthcare workers towards FPDR and determine the facilitating factors and barriers regarding its systemic implementation into clinical practice [5]. Many HCPs reported negative attitudes towards FPDR, often due to concerns regarding adverse effects on patient outcome, distraction to resuscitation team and interference to the resuscitation efforts, breach of patient’s privacy, misinterpretation of the situation, malpractice litigation, difficulty stopping futile CPR, addition of performance anxiety and stress on resuscitation team, and psychological trauma to the family members having witnessed the resuscitation process [4,5,12,13,16-19]. Additionally, family members may not fully understand the medical procedures and interventions taking place, which can lead to confusion, anxiety, misunderstanding, and anger [9].

Healthcare workers' readiness to include family during resuscitation is crucial for allowing family presence. An FPDR policy must consider potential issues and gain acceptance from all involved before implementation [7].

A 2008 study in Malaysia found only 15.8% of emergency department HCPs supported FPDR [20]. In contrast, a 2011 study showed 76.1% of the public favoured FPDR [21], highlighting a significant perception gap between HCPs and the general public. To date, the stance of ICU practitioners in Malaysia on the practice of FPDR remains unknown.

Currently, there is no established culture or written policy for implementing FPDR in our setting. The decision to include or exclude family members during resuscitation is left to the doctors leading the effort. The perception of Malaysian critical care HCPs towards FPDR has largely remained unassessed to this date. Our study aims to explore the current attitudes and willingness of doctors in critical care settings towards FPDR and to identify the factors that either hinder or support this practice in our local context.

Therefore, the primary objective of this study was to explore the attitudes and willingness of doctors in Malaysian critical care settings towards FPDR and to identify the factors influencing their views.

## Materials and methods

This cross-sectional study collected opinions of doctors working in critical care wards of Hospital Kuala Lumpur (HKL) and Hospital Canselor Tuanku Muhriz UKM (HCTM) towards the practice of FPDR from October 2023 to January 2024. HKL is the central national tertiary referral centre under the Ministry of Health, while HCTM is a prominent public university teaching hospital.

The study received approval from the Research Committee of the Department of Anaesthesiology & Intensive Care, Faculty of Medicine Universiti Kebangsaan Malaysia (UKM), Research Ethics Committee UKM (Ref No: UKM PPI/111/8/JEP-2023-505 & UKM.FPR.SPI 800-2/27), Medical Research & Ethics Committee (MREC), and the National Medical Research Registry (NMRR) (NMRR ID: NMRR ID-23-02038-TW9 & Research ID: RSCH ID-23-02532-DK9).

In this study, we used a validated questionnaire titled “Physicians’ Opinion and Attitude Regarding Presence of Family Members During Invasive Medical Procedures and Resuscitation” by Al Bshabshe et al. [12]. This self-administered questionnaire was designed using different theories, including health belief models, programmed behaviour, the theory of reasoned action, and the theory of self-efficacy. The aspects discussed in each theory were combined to formulate the questionnaire, and its validity was tested in many studies [12]. Permission has been obtained from Dr. Ali A. Al Bshabshe of King Khalid University, Saudi Arabia, to use the questionnaire in this study [12].

The questionnaire consists of two sections. The first section consists of demographic data of the respondents without identifying data. The respondents were also asked about their previous exposure, training, and experience with FPDR. The second section includes 27 questions designed in the form of either four-point Likert scales or multiple-choice questions to explore the opinions of healthcare professionals on the practice of FPDR. The Likert scale consists of four options: totally agree (1), agree (2), disagree (3), and totally disagree (4). The participating doctors’ beliefs and opinions pertaining to FPDR were explored, including perceived benefits and drawbacks of FPDR in their daily work. The self-efficacy to handle such situations and their associated social pressure was explored. The respondents were initially asked whether they agree that family should be allowed to be present during the process of a resuscitation, followed by their opinions regarding potential immediate or delayed undesirable effects (nine questions) and desirable consequences of FPDR (seven questions). As described in the original methodology by Al Bshabshe et al., the questionnaire concludes by asking the physician whether he or she would allow FPDR in their practice. For this study, the primary outcome of "willingness" was defined by the response to this question, thereby measuring a physician's self-reported intention to perform this clinical behaviour.

In this study, we contacted anaesthesia and critical care doctors who manage patients in the intensive care units (ICUs) of HKL and HCTM through emails using the email database of both institutions from October 2023 to January 2024. The invitation email contained the information about our study, informed consent, and a link to an online FPDR questionnaire by Al Bshabshe et al. [12]. The inclusion criteria were doctors working in the ICU, including service medical officers, trainees of anaesthesiology and intensive care, anaesthesiologists, consultant anaesthesiologists, and intensivists of both institutions. Doctors with less than six months of experience working in the ICU were excluded from the study.

Sample size was calculated using the Krejcie & Morgan formula [22] for a finite population where the population size is 187. The largest sample size is based on Al Bshabshe et al.'s study, which indicated 47.9% of healthcare workers had agreement towards difficulty discontinuing failed CPR [12]; therefore, 160 subjects were the minimum sample size to be recruited with 80% power of the study, 95% confidence level, and anticipation of a 20% dropout rate.

## Results

A total of 187 invitation emails were sent out, with 169 responses received. Ten respondents were excluded from this study because they had less than six months of working experience in the ICU, making a total of 159 (85.0%) responses. We received 101 (63.5%) respondents from HKL and 58 (36.5%) respondents from HCTM. The majority of respondents (81.8%, n = 130) reported that they did not receive any education concerning FPDR. Only a total of 18.2% (n = 29) of them received information regarding the subject in continuing medical education (CME) or resuscitation courses. Their demographic and characteristic data are shown in Table 1.

**Table 1:** Characteristics of the respondents. Data are expressed in frequency (%). ICU: intensive care unit; FPDR: family presence during resuscitation.

Variables		Subjects (N = 159), n (%)
Age group (years)	25-29	3 (1.9)
	30-34	84 (52.8)
	35-39	51 (32.1)
	40-49	18 (11.3)
	50 and above	3 (1.9)
Gender	Female	103 (64.8)
	Male	56 (35.2)
Ethnicity	Malay	72 (45.3)
	Chinese	74 (46.5)
	Indian	10 (6.3)
	Others	3 (1.9)
Religion	Islam	74 (46.5)
	Christianity	18 (11.3)
	Buddhism	49 (30.8)
	Hinduism	8 (5)
	Others	10 (6.3)
Workplace	Hospital Kuala Lumpur (HKL)	101 (63.5)
	Hospital Canselor Tuanku Muhriz UKM (HCTM)	58 (36.5)
Professional level	Medical officer	31 (19.5)
	Postgraduate trainees in anaesthesiology and critical care	100 (62.9)
	Specialist	23 (14.5)
	Consultant	5 (3.1)
Experience in the ICU	6 months – 2 years	12 (7.5)
	2 – 5 years	61 (38.4)
	>5 years	86 (54.1)
Received information regarding FPDR	No	130 (81.8)
	Yes	29 (18.2)

A total of 34 (21.4%) respondents had experience with FPDR before, either as a team leader or member, and further analysis of these respondents according to their workplaces did not show any statistically significant difference between the two hospitals. Table 2 depicts the experiences with FPDR from both hospitals.

**Table 2:** Experience with FPDR according to the workplace. Data are expressed in frequency (%). P-value by the chi-square test. FPDR: family presence during resuscitation.

Workplace	Done FPDR				
	No	Yes	P-value	df	Effect size (Cramer's V)
Hospital Kuala Lumpur (HKL)	80 (20.8)	21 (79.2)	0.810	1	0.019
Hospital Canselor Tuanku Muhriz UKM (HCTM)	45 (32.8)	13 (67.2)			

Respondents’ views about the potential effects of FPDR are shown in Table 3. Overall, the majority of respondents (93.9%) agreed that allowing FPDR may increase stress on medical staff. Concerns were also raised regarding the potential for family members to become emotionally distressed and interfere with the resuscitation process (94.5%). Most agreed that allowing family presence during active resuscitation provides an opportunity to educate the family regarding the patient's condition (63.5%), and it helps to remove family doubts regarding the best efforts offered to the patient (64.8%). The presence of family was also thought to encourage the professionalism of staff during resuscitation (73.6%). Opinions were fairly split regarding FPDR improving family participation in patient care, family support to the staff, and family bonding. On the other hand, there was substantial disagreement with the assumption that FPDR can reduce the fear and anxiety of family members (74.9%).

**Table 3:** Respondents’ view about the effects of FPDR. Data are expressed in frequency (%). CPR: cardiopulmonary resuscitation; FPDR: family presence during resuscitation.

	Participants’ response			
Respondents’ view	Totally agree, n (%)	Agree, n (%)	Disagree, n (%)	Totally disagree, n (%)
Assumed potential undesirable effects				
Misinterpretation of procedures during active resuscitation	71 (44.7)	64 (40.3)	17 (10.7)	7 (4.4)
Decreased bedside space for staff	75 (47.2)	53 (33.3)	15 (9.4)	16 (10.1)
Breach of privacy and confidentiality	64 (40.3)	50 (31.4)	38 (23.9)	7 (4.4)
Increase stress on staff	88 (55.5)	61 (38.4)	7 (4.4)	3 (1.9)
Interference by family members with patient care	85 (53.5)	65 (40.9)	6 (3.8)	3 (1.9)
Difficulty continuing futile CPR	64 (40.3)	55 (34.6)	31 (19.5)	9 (5.7)
Assumed desirable effects				
Help educate the family about the patient’s condition	36 (22.6)	65 (40.9)	43 (27.0)	15 (9.4)
Improve the family participation of the patient’s family in patient care	23 (14.5)	61 (38.4)	54 (34)	21 (13.2)
Improve family support for the staff	18 (11.3)	60 (37.3)	61 (38.4)	20 (12.6)
Remove family doubts about care	35 (22.0)	68 (42.8)	38 (23.9)	18 (11.3)
Reduce family anxiety and fear	11 (29.0)	29 (18.2)	79 (49.7)	40 (25.2)
Improve family bonding with the patient	16 (10.1)	56 (35.2)	57 (35.8)	30 (18.9)
Increase staff professionalism	44 (27.7)	73 (45.9)	28 (17.6)	14 (8.8)

Respondents also generally view that FPDR will increase the risk of psychological traumas to the family (74.8%, n = 119), stress on the medical team (85.5%, n = 136), and family raising complaints or litigation against the staff (66%, n = 105). These data are shown in Table 4.

**Table 4:** Respondents’ view about the potential delayed effects of FPDR. Data are expressed in frequency (%). FPDR: family presence during resuscitation.

Assumed delayed effects	Doctors’ response		
	No effect, n (%)	Decrease risk, n (%)	Increase risk, n (%)
Psychological trauma to family members	19 (11.9)	21 (12.2)	119 (74.8)
Professional stress among staff	21 (13.2)	2 (1.3)	136 (85.5)
Malpractice litigation against the staff	25 (15.7)	29 (18.2)	105 (66.0)

A total of 115 (72.3%) of the respondents indicated that they would not allow FPDR in their daily work, while only 44 (27.7%) would allow the practice of FPDR. There were no association of respondents’ demographic and characteristic data with their willingness to practice FPDR, except for factors including “received information regarding FPDR” and “experience in FPDR”. Using simple logistic regression, we found that those who did not receive information regarding FPDR (OR: 3.732 (1.615-8.620), p = 0.002) and those without experience in FDPR (OR: 10.355 (4.391-24.421), p < 0.001) were significantly different. This is shown in Table 5.

**Table 5:** Association of respondents’ characteristics and willingness to allow FPDR. Data are expressed in frequency (%) or median (25th-75th percentile). ^a ^P-value by the Pearson chi-square test. * P-value by Fisher's exact test (used when >20% of cells had an expected count <5). ^b ^P-value for simple logistic regression. ^c^ Degrees of freedom and Cramer's V are not applicable for age (years), as its association with willingness to allow FPDR was analysed using a Mann-Whitney U test, not a chi-square test. ICU: intensive care unit; CPR: cardiopulmonary resuscitation; FPDR: family presence during resuscitation.

		Would you allow family presence during CPR?		P-value^a^	df	Effect size (Cramer's V)	Odd ratio (95% CI)	P-value^b^
		No, (n = 115)	Yes, (n = 44)					
Age (years)		34.0 (33.0-37.0)	34.5 (32.0-36.8)	0.934	- ^c^		1.006 (0.928 – 1.091)	0.878
Gender	Female	75 (65.2)	28 (63.6)	0.855	1	0.015	0.933 (0.452 – 1.926)	0.852
	Male (ref.)	40 (34.8)	16 (36.4)					
Ethnicity	Malay (ref.)	49 (42.6)	23 (52.3)	0.230*	3	0.160		
	Chinese	57 (49.6)	17 (38.6)				0.635 (0.305 – 1.324)	0.226
	Indian	8 (7.0)	2 (4.5)				0.533 (0.105 – 2.710)	0.448
	Others	1 (0.9)	2 (4.5)				4.261 (0.367 – 49.428)	0.246
Religion	Islam (ref.)	51 (44.3)	23 (52.3)	0.856*	4	0.163		
	Christianity	14 (12.2)	4 (9.1)				0.634 (0.188 – 2.136)	0.462
	Buddhism	36 (31.3)	13 (29.5)				0.801 (0.359 – 1.787)	0.587
	Hinduism	7 (6.1)	1 (2.3)				0.317 (0.037 – 2.726)	0.295
	Others	7 (6.1)	3 (6.8)				0.950 (0.225 – 4.008)	0.945
Workplace	Hospital Kuala Lumpur (HKL)	75 (65.2)	26 (59.1)	0.473	1	0.057	0.770 (0.378 – 1.572)	0.473
	Hospital Canselor Tuanku Muhriz UKM (HCTM) (ref.)	40 (34.8)	18 (40.9)					
Professional level	Medical officer (ref.)	21 (18.3)	10 (22.7)	0.864*	3	0.075		
	Trainee	72 (62.6)	28 (63.6)				0.817 (0.342 – 1.950)	0.648
	Specialist	18 (15.7)	5 (11.4)				0.583 (0.168 – 2.025)	0.396
	Consultant	4 (3.5)	1 (2.3)				0.525 (0.052 -5.327)	0.586
Received info regarding FPDR before	No (ref.)	101 (87.8)	29 (65.9)	0.001	1	0.254		
	Yes	14 (12.2)	15 (34.1)				3.732 (1.615 – 8.620)	0.002
Experienced FPDR	No (ref.)	104 (90.4)	21 (47.7)	<0.001	1	0.466		
	Yes	11 (9.6)	23 (52.3)				10.355 (4.391 – 24.421)	<0.001
ICU Experience	6 months – 2 years (ref.)	10 (8.7)	2 (4.5)	0.660	2	0.072		
	2 – 5 years	43 (37.4)	18 (40.9)				2.093 (0.416 – 10.522)	0.370
	>5 years	62 (53.9)	24 (54.5)				1.935 (0.395 – 9.488)	0.416

Using a multivariable model, we further analysed the two significant factors and found that previous experience in FPDR is significantly associated with respondents’ willingness to allow FPDR (OR: 8.823 (95% CI: 3.670-21.212), p < 0.001). However, having received information regarding FPDR beforehand is not found to be significantly associated (OR: 2.411 (95% CI: 0.927-6.270), p = 0.071).

We also looked into the association between respondents' opinions regarding the effects of FPDR and their willingness to allow it. We grouped these opinions into two groups of “agree” and “disagree” for ease of analysis. Those who would not allow FPDR significantly agreed to all of the undesirable effects of FPDR except the factor “increase stress on staff” (Table 6). These findings suggest that such factors represent significant barriers to doctors' acceptance of FPDR.

**Table 6:** Association of respondents’ opinions regarding undesirable effects of FPDR with the willingness to allow FPDR. Data are expressed in frequency (%). P-value by Pearson chi-square test. * P-value by Fisher's exact test (used when >20% of cells had an expected count <5). CPR: cardiopulmonary resuscitation; FPDR: family presence during resuscitation.

Assumed potential undesirable effects		Would you allow family presence during CPR?		P-value	df	Effect size (Cramer's V)
		No (n = 115)	Yes (n = 44)			
Misinterpretation of procedures during active resuscitation	Agree	108 (93.9)	27 (61.4)	<0.001*	1	0.407
	Disagree	7 (6.1)	17 (38.6)			
Decreased bedside space for staff	Agree	98 (85.2)	30 (68.2)	0.015*	1	0.192
	Disagree	17 (14.8)	14 (31.8)			
Breach of privacy and confidentiality	Agree	93 (80.9)	21 (47.7)	<0.001	1	0.329
	Disagree	22 (19.1)	23 (52.3)			
Increase stress on staff	Agree	110 (95.7)	39 (88.6)	0.141*	1	0.129
	Disagree	5 (4.3)	5 (11.4)			
Interference by family members with patient care	Agree	113 (98.3)	37 (84.1)	0.002*	1	0.274
	Disagree	2 (1.7)	7 (15.9)			
Difficulty continuing futile CPR	Agree	99 (86.1)	20 (45.5)	<0.001	1	0.419
	Disagree	16 (13.9)	24 (54.5)			

Respondents who were not willing to allow FPDR also had the opinion that FPDR significantly increases the risk for causing psychological trauma to family members, increases the risk for stress among staff, and increases the risk of malpractice/litigation against staff (Table 7). Conversely, respondents who perceived FPDR as having desirable effects were significantly willing to allow FPDR, as shown in Table 8. These findings underscore the influence of perceived risks and benefits on doctors' acceptance of FPDR.

**Table 7:** Association of respondents’ opinion regarding delayed effects of FPDR with the willingness to allow FPDR. Data are expressed in frequency (%). P-value by Pearson chi-square test. * P-value by Fisher's exact test (used when >20% of cells had an expected count <5). CPR: cardiopulmonary resuscitation; FPDR: family presence during resuscitation.

Assumed delayed effects		Would you allow family presence during CPR?		P-value	df	Effect size (Cramer's V)
		No (n = 115)	Yes (n = 44)			
Psychological trauma to family members	Decrease risk	14 (12.2)	7 (15.9)	<0.001	2	0.306
	No effect	7 (6.1)	12 (27.3)			
	Increase risk	94 (81.7)	25 (56.8)			
Professional stress among staff	Decrease risk	0 (0)	2 (4.5)	<0.001*	2	0.431
	No effect	6 (5.2)	15 (34.1)			
	Increase risk	109 (94.8)	27 (61.4)			
Malpractice litigation against the staff	Decrease risk	9 (7.8)	20 (45.5)	<0.001	2	0.456
	No effect	17 (14.8)	8 (18.2)			
	Increase risk	89 (77.4)	16 (36.4)			

**Table 8:** Association of respondents’ opinion regarding desirable effects of FPDR with the willingness to allow FPDR. Data are expressed in frequency (%). P-value by Pearson chi-square test. * P-value by Fisher's exact test (used when >20% of cells had an expected count <5). CPR: cardiopulmonary resuscitation; FPDR: family presence during resuscitation.

Assumed desirable effects		Would you allow family presence during CPR?		P-value	df	Effect size (Cramer's V)
		No (n = 115)	Yes (n = 44)			
Help educate the family about the patient’s condition	Agree	58 (50.4)	43 (97.7)	<0.001	1	0.440
	Disagree	57 (49.6)	1 (2.3)			
Improve the family participation of the patient’s family in patient care	Agree	48 (41.7)	36 (81.8)	<0.001	1	0.359
	Disagree	67 (58.3)	8 (18.2)			
Improve family support for the staff	Agree	46 (40)	32 (72.7)	<0.001	1	0.293
	Disagree	69 (60)	12 (27.3)			
Remove family doubts about care	Agree	61 (53)	42 (95.5)	<0.001	1	0.397
	Disagree	54 (47)	2 (4.5)			
Reduce family anxiety and fear	Agree	18 (15.7)	22 (50)	<0.001	1	0.354
	Disagree	97 (84.3)	22 (50)			
Improve family bonding with the patient	Agree	38 (33)	34 (77.3)	<0.001	1	0.398
	Disagree	77 (67)	10 (22.7)			
Increase staff professionalism	Agree	77 (67)	40 (90.9)	0.002	1	0.243
	Disagree	38 (33)	4 (9.1)			

## Discussion

Demographic characteristics and FPDR acceptance

In this cross-sectional study, 72.3% (n = 115) of respondents opposed FPDR. This finding was similar to a study by Badir et al. (2007), who surveyed respondents from seven major hospitals in Turkey and found that most were unfamiliar with FPDR and the majority did not agree that allowing FPDR was necessary [23]. However, in the United States, Maclean et al. (2003), who conducted a similar survey among critical care and emergency HCPs, found that many had brought family members to the bedside for resuscitation [15]. These studies have shown a significant contrast across different cultural contexts. In a local context, a multi-centre study involving Hospital Kuala Lumpur (HKL), Hospital Pulau Pinang (HPP), Hospital Universiti Sains Malaysia (HUSM), and Universiti Malaya Medical Centre (UMMC) demonstrated that the concept of FPDR is not well accepted among Malaysian healthcare professionals, with only 15.8% of emergency healthcare staff agreeing to allow FPDR in their practice [20].

We surveyed HCPs from two tertiary hospitals in Kuala Lumpur. HKL is a 2300-bedded hospital under the Ministry of Health, Malaysia, while HCTM is a 706-bedded teaching hospital of Universiti Kebangsaan Malaysia. There was no notable difference in the acceptance of FPDR between these two hospitals. This is consistent with surveys conducted in other teaching and general hospitals across Asian countries. Notably, our study revealed that demographic characteristics, such as age, gender, diverse cultural backgrounds of ethnicity, and religion, did not influence the acceptance of FPDR. Kianmehr et al. (2010), in a comparable study conducted in four teaching hospitals within a predominantly Muslim environment in Iran, also found that demographic factors, including gender and age, did not predict opinions towards FPDR. Similarly, the common reasons cited for opposition to family presence included fears of psychological trauma to family members, potential interference with patient care and decision-making, and a perceived increase in staff stress [24]. This is supported by another survey conducted at the General Hospital of Singapore by Ong et al. (2014), which found that respondents were generally not in favour of witnessed resuscitation [25]. The study noted that the most common reasons for not wanting relatives present included concerns that witnessing the resuscitation process would be a traumatic experience for them, the possibility that relatives might ask too many questions and interfere with the resuscitation, the potential for relatives to cause stress for the staff performing the procedure, and the potential risk of medico-legal issues arising [25].

Professional levels (including medical officers, trainees, specialists, and consultants) and ICU experience did not significantly correlate with FPDR acceptance in our study. This contrasted with the findings of Chapman et al. (2012) from Australia, which indicated that staff with speciality certification and higher qualifications were more likely to perceive benefits and fewer risks associated with FPDR. They also demonstrated that staff with speciality certification, who were more senior and reported a greater duration of experience in their role, were significantly more likely to exhibit greater self-confidence in their ability to manage FPDR [18]. This implies that factors related to professional training, experience, and institutional setting may not be the sole drivers of attitudes towards FPDR.

The impact of information and experience

Previous experience with FPDR was the most significant factor influencing a doctor's willingness to allow the practice.

The study highlights the significant role of information and experience in shaping doctors' opinions on FPDR. Those who had received information about FPDR before the survey were more likely to accept the practice, suggesting that knowledge and understanding are crucial factors in influencing attitudes. This finding is consistent with previous research by Meghani et al., indicating that educating doctors on FPDR protocols and benefits can positively impact their attitudes and practices [10]. Understanding the potential advantages of FPDR for families and patients can help HCPs feel more comfortable implementing it.

Furthermore, respondents with personal experience of FPDR were significantly more inclined to support its implementation (p < 0.001), demonstrating a strong association (Phi = 0.466) with willingness to allow FPDR (details in Table 5). This indicates that first-hand exposure to the FPDR practice can foster a more positive perception of its potential benefits and challenges. This finding aligns with the study by Axelsson et al. (2005), who noted that those who had participated in an FPDR programme believed that FPDR was not only beneficial for the family but also for the staff [7]. Observing positive outcomes or witnessing how families cope with being present during resuscitation could shape their perspective and encourage them to offer this option to future families.

Perceived undesirable effects of FPDR

A majority of the respondents who are not willing to allow FPDR perceived that the practice has undesirable effects, such as potential misinterpretation of the resuscitation process by family members, increasing staff stress, insufficient physical space, privacy violations, emotional distress for family members, difficulty for HCPs to discontinue futile resuscitation, and fear of increased risk of complaints and litigation.

Witnessing chest compressions, defibrillator shocks, and various medical equipment can cause significant distress for family members in an already emotional state. The presence of distressed family members could also create emotional strain for the medical team, potentially impeding clear communication and efficient execution of resuscitation procedures. However, it is important to acknowledge that studies by far have not shown a negative impact on resuscitation quality due to FPDR [4]. A study comparing 252 hospitals with and without FPDR policy showed that there was no statistically significant difference in outcomes and processes of care when the outcomes of return of spontaneous circulation and survival to discharge were measured [26].

Many participants believed that FPDR increases the risk of adverse psychological trauma for family members. This aligns with several studies in which healthcare providers express concern about the psychological impacts on families [12,27]. However, Oczkowski et al. (2015) [13] and Jabre et al. (2013) [4] have presented contradictory findings, showing that FPDR can improve the psychological outcomes for family members who witnessed CPR, resulting in fewer symptoms of anxiety, depression, post-traumatic stress disorder (PTSD), and complicated grief compared to those who do not witness the procedure. Many family members felt that their presence might ease the suffering of their loved ones and that being with the deceased in their final moments aided their grieving and adjustment to death, as evidenced by studies conducted by Doyle et al. (1987), Axelsson et al. (2005), and Alshaer et al. (2018) [2,7,28]. Witnessing the resuscitation provides assurance that everything possible has been done and offers closure and healing to the family when death becomes inevitable [5,13].

Many healthcare workers have expressed concerns about violating the patient’s privacy and confidentiality if FPDR is allowed. Active resuscitation inevitably involves exposure of areas of the body to facilitate resuscitation actions, and many procedures appear somewhat aggressive, if not violent, to non-medical personnel. A survey of 200 individuals, including patients and their family members in the emergency department's waiting room, revealed that most preferred having a family member present during resuscitation. However, more than half indicated that they wanted only a specific family member to be present, and their preferences varied [29]. On the other hand, reports from survivors of cardiac arrest indicated patients’ willingness to have family members at the bedside and did not consider this a violation of their privacy [30]. Nevertheless, it is crucial to respect a patient's autonomy and honour their documented wishes in these situations. It is important to consider a patient's existing advance directive when making the decision to allow FPDR.

Another practical concern arising from FPDR is the constraint of space. A significant portion of respondents who opposed FPDR expressed that decreased bedside space was a concern. However, interestingly, a substantial number of respondents who support FPDR also recognise this potential issue. This suggests that while bedside space constraints are a valid and practical concern, they may not be the primary factor in determining a doctor's stance on FPDR.

Regardless of their stance on FPDR, a large majority of respondents believed that family presence could increase staff stress. The stress may arise from various factors, such as performance anxiety in front of onlookers, feeling pressured to manage the family's emotions while focusing on life-saving interventions, and anticipating distractions from distressed family members or complaints arising from misunderstandings. This can be particularly challenging when the resuscitation attempts are unsuccessful [7]. This suggests that, while it might not be an impeding factor to acceptance of FPDR, addressing staff well-being is a critical consideration when implementing FPDR.

Hospital policies must facilitate communication between family and medical teams, helping them understand the resuscitation process and minimising interruptions from family members despite emotions. Implementing a standardised policy helps reduce variations in medical practices across departments and hospitals, eliminating stress on clinicians forced to make difficult decisions regarding family presence during clinical deterioration [9].

Perceived desirable effects of FPDR

Respondents’ opinions on the potential benefits of FPDR were explored, including education opportunities, facilitating family involvement in care, improving family support, removing family doubts and reducing their anxiety, strengthening patient-family relationships, and encouraging professional behaviour among staff.

There was evident divergence in the response regarding improved family facilitation in patient care, support for families and staff, and the strengthening of patient-family relationships. Respondents may harbour concerns about the emotional burden FPDR might place on families witnessing resuscitation. This could also explain the disagreement surrounding the reduction of fear and anxiety. Some respondents believe that FPDR exacerbates these emotions, while others consider the presence of the family to be a source of comfort for both the patient and their loved ones. This view is consistent with studies showing that some families reported that their presence could alleviate the patient's suffering and was beneficial for both the patient and family members [2,28].

Most would agree that allowing FPDR create opportunities to educate the family about the patient’s condition. FPDR provides families with a firsthand understanding of the severity of their loved one's condition and the efforts being made to save their life [5,15]. This can help alleviate uncertainty and confusion and foster a more open and honest dialogue between healthcare providers and families [28]. Witnessing the resuscitation process can empower families to make informed decisions about end-of-life care, should the need arise [15].

A strong consensus emerged among respondents on two key aspects. They overwhelmingly perceived FPDR as a means of reassuring families that everything possible is being done for the patient. It provides an opportunity to explain medical procedures, answer questions, and address concerns in real time, potentially fostering trust and minimising doubts later on. Furthermore, they concurred that it promotes a professional demeanour among staff, possibly due to the increased awareness of being observed by family members.

Respondents who acknowledged the desirable effects of FPDR were found to be more likely to support its implementation in their practice despite mixed responses regarding some of the desirable outcomes explored in this study.

This survey underscores the multifaceted nature of FPDR in the ICU. While the educational benefits for the family and promotion of staff professionalism are recognised, the emotional impact on families and the medical team remains a primary concern for respondents. Findings highlight the significance of education and first-hand experience in influencing respondents' viewpoints on FPDR. To encourage wider FPDR implementation, interventions should focus on addressing current concerns through targeted education, FPDR-specific training, and the establishment of clear, local guidelines [10]. Future research should explore the optimal content of FPDR education and investigate the factors that contribute to positive FPDR experiences for both doctors and families. Additionally, in-depth interviews with doctors who have witnessed FPDR could provide valuable insights into the factors that shaped their positive experiences.

The idea of FPDR embodies a triangular dynamic, wherein the involvement of family members impacts the healthcare professional, the present relatives, and the patient's care. It is imperative to harmonise the needs and well-being of all these parties within the FPDR framework, given that each group's actions can influence others [1]. Further research into the psychological effects on families and staff could shape future ICU protocols for FPDR. This may involve in-depth interviews with affected families or studies tracking psychological well-being before, during, and after resuscitation events with family presence. By gaining a deeper understanding of the emotional aspects of FPDR, we can create more comprehensive ICU protocols that balance the needs of patients, families, and medical professionals.

The current lack of clear guidelines may cause HCPs to be confused and hesitant in allowing FPDR in their practice. A well-defined policy detailing the process for FPDR, including eligibility criteria, communication protocols with families, staff training procedures, and staff responsibilities, can provide HCPs with a roadmap for handling these complex situations [3,5]. This can create a sense of reassurance and prepare them to make well-informed decisions about FPDR on a case-by-case basis [5,15]. By outlining a standardised approach, the policy also ensures that all families are offered the option of FPDR fairly and consistently [5]. This could improve communication with families during critical situations and potentially enhance patient-centred healthcare.

Limitations

Despite its valuable insights into doctors' opinions on FPDR in Kuala Lumpur, this study possesses several limitations that warrant consideration. The reliance on a self-administered online questionnaire rendered the study susceptible to response bias, particularly social desirability bias, where participants may have provided responses perceived as more socially acceptable rather than reflecting their genuine opinions. Furthermore, this methodology captures self-reported perceptions and attitudes rather than actual clinical outcomes or behaviours. The stated willingness to allow FPDR, for instance, may not perfectly correlate with actions taken during a real-world resuscitation event.

Furthermore, the study was conducted exclusively in two major tertiary hospitals in Kuala Lumpur. While these institutions represent significant critical care environments within the region, the findings may not be fully generalizable to medical practitioners in other Malaysian critical care settings, including smaller district or private facilities, nor to other healthcare professionals involved in resuscitation, such as nurses or paramedics. This limitation is underscored by the significant influence that diverse cultural and institutional contexts can exert on attitudes towards practices like FPDR. As resuscitation is fundamentally a multidisciplinary team effort, the focus solely on physicians means our findings do not capture the perspectives of other vital team members. The views of nurses, who often have the most direct and sustained contact with families, are particularly important and represent a key area for future investigation.

A further limitation pertains to the statistical precision of our findings. Several parameters in our analysis were associated with wide 95% confidence intervals, suggesting a considerable degree of uncertainty around the point estimates. This lack of precision is likely attributable to the study's relatively modest sample size (N = 159). Consequently, the results should be interpreted with caution, and future research with larger samples is warranted to produce more precise estimates and allow for more robust conclusions.

Lastly, the questionnaire's predefined scope might not have comprehensively captured all nuances inherent in doctors' perspectives. Although the survey explored various perceived benefits and drawbacks, the intricate complexity of attitudes towards FPDR could involve additional unmeasured factors. Future qualitative research, employing methods such as in-depth interviews, would be beneficial to explore these complexities more thoroughly.

## Conclusions

This study revealed that the majority of respondents working in critical care settings in HKL and HCTM are opposed to FPDR. Their reluctance is due to the perceived negative effects of FPDR. Experience in FPDR was the factor that significantly influenced the willingness to practise FPDR. Crucially, the study found that personal experience with FPDR significantly influenced a doctor's willingness to practice it, suggesting that direct exposure may foster a more positive perception. These findings highlight the need for targeted education and the development of clear, supportive guidelines to address concerns and potentially encourage wider acceptance and implementation of FPDR in Malaysian critical care settings.

## Appendices

**Table 9:** Data collection sheet: Demographic. CME: continuing medical education. Source: Al Bshabshe et al. [12]. Permission to use the questionnaire in this study has been obtained from Dr. Ali A. Al Bshabshe of King Khalid University, Saudi Arabia.

Demographics		
Age (years)		
Gender	Male/Female	
Ethnicity	Malay/Chinese/Indian/Others (please specify)	
Religion	Islam/Hindu/Christian/Buddhist/Others (please specify)	
Workplace	Hospital Kuala Lumpur (HKL)/Hospital Canselor Tuanku Muhriz (HCTM)	
Job description	Consultant	
	Specialist	
	Medical officer	
Experience working in an adult general ICU	< 6 months, 6 months – 2 years, 2 – 5 years, > 5 years	
Academic year (if master's trainee in anaesthesiology and critical care)	Year 1, Year 2, Year 3, Year 4	
Received information regarding family presence during resuscitation before (during CME/resuscitation courses)	Yes (please specify), No	
As a doctor or team leader of resuscitation, have you ever given the option to family members to witness the cardiopulmonary resuscitation (CPR) process?	Yes, No	
As part of a team member during resuscitation, have you experienced the team leader giving the option to a family member to witness or be at the patient’s bedside during the CPR process?	Yes, No	

**Table 10:** Data collection sheet: Opinion. CPR: cardiopulmonary resuscitation. Source: Al Bshabshe et al. [12]. Permission to use the questionnaire in this study has been obtained from Dr. Ali A. Al Bshabshe of King Khalid University, Saudi Arabia.

Item	Response			
1. Family member(s) should be present during resuscitation. What do you think?	1. Totally agree	2. Agree	3. Disagree	4. Totally disagree
2. Family member(s) should be present during cardiac arrest. What do you think?	1. Totally agree	2. Agree	3. Disagree	4. Totally disagree
3. Family member(s) should be present during an invasive medical procedure. What do you think?	1. Totally agree	2. Agree	3. Disagree	4. Totally disagree
4. What do you think will be the impact of family member(s)' presence during resuscitation?	a. They will be prone to having a higher risk of psychological traumas in the future, like post-traumatic stress disorder, depression, and anxiety.	b. Their chance of having psychological trauma like post-traumatic stress disorder, depression, and anxiety will decrease.	c. It will not make any difference in their risk of developing psychological trauma like post-traumatic stress disorder, depression, and anxiety.	
5. Presence of family member(s) during resuscitation of their relatives will:	a. Increase the stress on the medical team.	b. Decrease the stress on the medical team.	c. Have no impact in this regard.	
6. Presence of family member(s) during the resuscitation of their relative will:	a. Decrease their anxiety at that moment.	b. Increase their anxiety at that moment.	c. Have no effect on them.	
7. Do you read any research about family presence during resuscitation?	a. Yes	b. No		
8. Presence of family member(s) during resuscitation will:	a. Increase the risk of complaint and litigation.	b. Decrease the risk of complaints and litigation.	c. No effect on complaint and litigation.	
9. If family member(s) is allowed to be present during resuscitation:	a. He/she may interfere with the process of resuscitation.	b. He/she may not interfere with the process of resuscitation.	c. His/her response will be very unpredictable in this regard.	
10. Do you think that the presence of family member(s) during resuscitation might lead to misinterpretation of some procedures during active resuscitation?	1. Totally agree	2. Agree	3. Disagree	4. Totally disagree
11. Do you agree with implementing a hospital policy regarding family presence during resuscitation?	1. Totally agree	2. Agree	3. Disagree	4. Totally disagree
12. Do you agree with implementing hospital policy regarding family member(s)' presence during invasive medical procedures?	1. Totally agree	2. Agree	3. Disagree	4. Totally disagree
13. If you have a relative who had cardiac arrest and the medical team is actively resuscitating him, you would prefer.	a. To be with him/her during the resuscitation process.	b. Not to be with him/her during the resuscitation process.	c. It would not matter whether or not to be with him/her during the resuscitation process.	
14. Do you think that the presence of family member(s) with their relative during resuscitation makes the space at the bedside insufficient for the resuscitation?	1. Totally agree	2. Agree	3. Disagree	4. Totally disagree
15. Do you think that the presence of family member(s) during resuscitation might violate the confidentiality and privacy of the patient?	1. Totally agree	2. Agree	3. Disagree	4. Totally disagree
16. Do you agree that in the presence of family member(s) during resuscitation, stress on staff may increase?	1. Totally agree	2. Agree	3. Disagree	4. Totally disagree
17. Do you agree that family member(s), if present during resuscitation, might lose emotional control and interfere with care?	1. Totally agree	2. Agree	3. Disagree	4. Totally disagree
18. Do you agree that the presence of family member(s) during resuscitation may make discontinuing resuscitation efforts difficult?	1. Totally agree	2. Agree	3. Disagree	4. Totally disagree
19. Do you think that the presence of family member(s) during resuscitation provides an opportunity to educate the family about the condition of the patient?	1. Totally agree	2. Agree	3. Disagree	4. Totally disagree
20. Do you agree that the presence of family member(s) during resuscitation may facilitate the participation of the patient’s family in caring for the patient?	1. Totally agree	2. Agree	3. Disagree	4. Totally disagree
21. Do you agree that the presence of family member(s) during resuscitation may improve the support from the family to the patient and the staff?	1. Totally agree	2. Agree	3. Disagree	4. Totally disagree
22. Do you agree that the presence of family member(s) during resuscitation may help remove doubt the family may have about care and reinforce their belief that everything possible was done?	1. Totally agree	2. Agree	3. Disagree	4. Totally disagree
23. Do you agree that the presence of family member(s) during resuscitation reduces fear and anxiety among the patient’s family member(s)?	1. Totally agree	2. Agree	3. Disagree	4. Totally disagree
24. Do you agree that the presence of family member(s) during resuscitation helps the patient-family relationship by improving connectedness and bonding with the patient?	1. Totally agree	2. Agree	3. Disagree	4. Totally disagree
25. Do you agree that the presence of family member(s) during resuscitation may encourage professional behaviour of staff at the bedside?	1. Totally agree	2. Agree	3. Disagree	4. Totally disagree
26. Would you allow family presence during CPR?	a. Yes	b. No		
27. Does family presence during resuscitation impact your clinical practice?	a. Yes	b. No		
